# MRI Insights in Chiari Malformation Type 1 and Variations With Hydrosyringomyelia

**DOI:** 10.7759/cureus.55676

**Published:** 2024-03-06

**Authors:** Anand M Hatgaonkar, Sandeep M Mahajan, Kajal A Hatgoankar, Gulshan R Bandre

**Affiliations:** 1 Radiodiagnosis, Datta Meghe Medical College, Datta Meghe Institute of Higher Education & Research (Deemed to be University), Nagpur, IND; 2 Radiodiagnosis, Government Medical College & Super Speciality Hospital, Nagpur, IND; 3 Pathology, Datta Meghe Medical College, Datta Meghe Institute of Higher Education & Research (Deemed to be University), Nagpur, IND; 4 Microbiology, Jawaharlal Nehru Medical College, Datta Meghe Medical College, Datta Meghe Institute of Higher Education & Research (Deemed to be University), Wardha, IND

**Keywords:** chiari malformations, chiari type 1 malformation, craniovertebral junction, syringomyelia, magnetic resonance imaging, tonsillar herniation, hydromyelia, cervical spine

## Abstract

Chiari malformation (CM) type 1 is a complex neurological disorder characterized by the displacement of the cerebellar tonsils into the upper spinal canal. Hydrosyringomyelia (HSM), which frequently coexists with this condition, presents diagnostic and treatment problems due to its broad spectrum of symptoms. There are various forms of CMs, with CM type 1 (CM1) being the most common type. Magnetic resonance imaging (MRI) is the best imaging technique to properly identify and diagnose CM1 and HSM. Important imaging findings include downward displacement of the cerebellar tonsils across the foramen magnum, the appearance of the syrinx in the spinal cord, and the alteration of the flow dynamics of the cerebrospinal fluid. This study was conducted at Datta Meghe Medical College, Nagpur, and Government Medical College & Super Speciality Hospital, Nagpur, India. It focuses on the diagnostic use of MRI in CM1 and its variations associated with HSM. Individuals who are asymptomatic may not need any treatment; however, those who are symptomatic or have HSM may require surgical decompression and restoration of the flow. We discuss the findings of MRI of six cases of CM1 and its variants with HSM and search for possible underlying causes. We conclude that magnetic resonance imaging is an imaging modality for the identification and evaluation of CM1 in cases of HSM.

## Introduction

Chiari malformations (CMs) represent complex neurological disorders characterized by the abnormal displacement of the cerebellar tonsils through the foramen magnum into the upper spinal canal. This condition, often congenital in nature, involves a structural anomaly where the lower part of the brain, specifically the cerebellum, descends into the upper spinal canal [1]. The condition was first described by Austrian pathologist Hans Chiari in the late 19th century. Since then, advancements in medical imaging techniques, such as magnetic resonance imaging (MRI), have enabled more accurate diagnosis and classification of CMs. There are several types of CMs, ranging from CM0 to CM5, each with its own specific characteristics and associated complications [2]. The most typical and mildest type of CM is CM1. CM0, CM0.5, and CM1.5 are considered variations of CM1 with or without hydrosyringomyelia (HSM). The other types of CMs are CM2, CM3, CM3.5, CM4, and CM5. All have different qualities and difficulties [3].

The development of syrinx is a complication that frequently accompanies CM1 [4]. Syrinxes are fluid-filled pockets that develop in the tissue of the spinal cord, and this condition is called syringomyelia. Hydromyelia is described as the dilation of the central canal of the spinal cord. These two conditions are also known as HSM as they cannot be distinguished separately [5]. Over time, these syrinxes can become larger, placing pressure on nearby spinal structures and causing a variety of neurological symptoms. Headache, neck pain, muscular weakness, sensory deficiencies, and breathing difficulty are a few of the symptoms that can range in severity from minor discomfort to neurological impairments [6,7]. Although the exact origin of CM1 and its connection to HSM are not yet known, it can be linked to cerebrospinal fluid (CSF) flow dynamics and developmental abnormalities throughout fetal development [8,9].

CM1 is diagnosed and evaluated using magnetic resonance imaging (MRI). Radiologists can precisely identify the abnormality and gauge its severity using comprehensive MRI images of the brain, cerebellum, spinal cord, and surrounding structures [10-14]. An appropriate MRI protocol is essential to capture detailed images of the brain and spinal structures.

Six cases of CM1 and its variants with HSM will be covered in this article. MRI findings will be evaluated and a search for possible underlying causes will be done based on clinical signs and symptoms. By learning more about CM1 with HSM, we seek to clarify this frequently misunderstood condition and offer insights into the best care strategies.

## Materials and methods

This study aimed to investigate the structural variations observed in CM1 patients with concurrent HSM using MRI. A retrospective analysis of MRI images was conducted to assess the anatomical features, severity, and associated abnormalities in CM1 patients with and without HSM.

This study was conducted simultaneously at Datta Meghe Medical College, Nagpur, and Government Medical College & Super Speciality Hospital, Nagpur, India. All newly diagnosed cases with CM1 and HSM, who underwent MRI between December 2022 and December 2023, for a period of one year attending tertiary care hospitals in central India were included in the study. Both pediatric and adult patients diagnosed with CM1 with concurrent HSM based on MRI findings were considered. Patients with a history of previous surgical interventions, incomplete MRI data, or poor image quality unsuitable for analysis were excluded from the study.

Informed written consent was obtained from the patients. In minor patients, consent was obtained from the parents or guardians. The institutional ethical committee approval was obtained. Patient confidentiality and data anonymization were ensured throughout the study process.

This study was carried out on a 1.5 Tesla MRI machine with a high-resolution, multi-sequence MRI protocol to capture detailed images of the brain and spinal structures. The T1-weighted, T2-weighted, T2-weighted gradient echo, and short tau inversion recovery (STIR) sequences were chosen. Images were obtained in multiple planes to visualize different tissue properties and anatomical structures. The T1-weighted and T2-weighted images were obtained in the axial and sagittal planes, T2-weighted gradient echo images were obtained in the sagittal plane, and STIR images were obtained in the sagittal and coronal planes. All patients of all ages and both sexes who underwent MRI cervical spine with or without MRI brain presenting with CM1 and HSM were chosen for this study. In some cases, whole-spine screening was performed with T2-weighted sequences in the sagittal plane. 

MRI findings were evaluated, and a search for possible underlying causes was done based on clinical signs and symptoms. By learning more about CM1 with HSM, we seek to clarify this frequently misunderstood condition and offer insights into the best care strategies.

## Results

A total of six cases were diagnosed with CM1 and HSM. Their ages ranged from eight to 34 years. Of the six patients, four were male and two were female (Table 1). These patients presented with a wide range of clinical symptoms, from headache and neck pain to sensory and motor deficits. Two patients came with complaints of decreased sensation of pain and temperature due to the syrinx. The MRI scan revealed downward displacement of the cerebellar tonsils across the foramen magnum and associated HSM (Table 1). The descent of the tonsils in the spinal canal ranged from four to 22 mm. Syrinx sizes also varied, from small, subcentimeter-sized to extensive, almost replacing the cord. All patients had CSF flow abnormalities with CSF flow artifacts.

**Table 1:** MRI findings in cases diagnosed with Chiari malformation type 1 and hydrosyringomyelia. MRI: magnetic resonance imaging, M: male; F: female

Case no.	Age (year)	Sex	MRI findings
1	31	F	MRI of the cervical spine with whole spine screening revealed: Displacement of the cerebellar tonsils by 12 mm. Associated hydrosyringomyelia extends from the C2-C3 to the D5-D6 intervertebral disc level.
2	34	M	MRI of the cervical spine with whole spine screening revealed: Displacement of the cerebellar tonsils by 11 mm. Associated hydrosyringomyelia extends from the C2 to D9 vertebral levels.
3	32	M	MRI of the brain and cervical spine revealed: Displacement of the cerebellar tonsils by 12 mm. Associated small syrinx extends from the C3 to C4 vertebral level.
4	08	F	MRI of the cervical spine with whole spine screening revealed: Displacement of the cerebellar tonsils by 9 mm. Associated hydrosyringomyelia extends from the C5 to D4 vertebral levels.
5	08	M	MRI of the cervical spine revealed: Displacement of the cerebellar tonsils by 4 mm. Extensive changes of hydrosyringomyelia involving the complete length of the spinal cord, extending from the cervico-medullary junction up to conus medullaris.
6	32	M	MRI of the brain revealed: Descent of the cerebellar tonsils into the upper spinal canal by 22 mm. Associated small syrinx at the level of the C2-C3 intervertebral disc level. Incidental finding of flattening of the skull base: Platybasia.

Case 1

**Figure 1:**
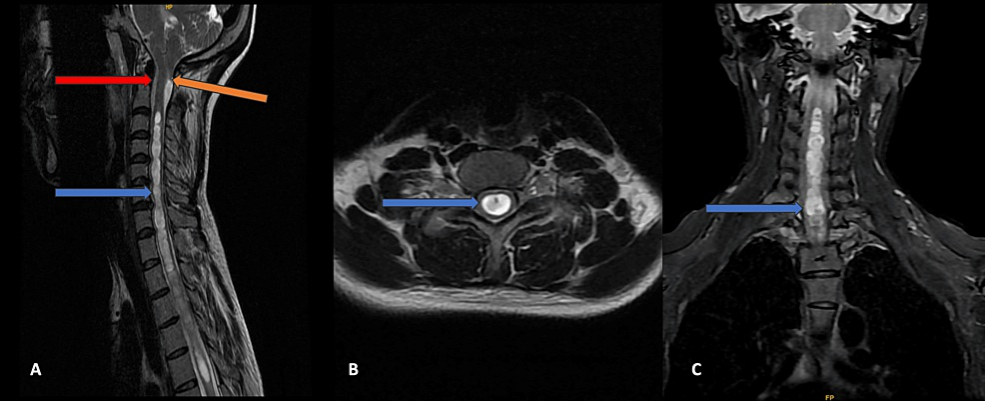
MRI of the cervical spine Magnetic resonance imaging (MRI) of the cervical spine, mid-sagittal T2-weighted image, shows tonsillar herniation (orange arrow), medullary kinking (red arrow), and hydrosyringomyelia (blue arrow) (A). Axial and coronal MRI T2-weighted images show hydrosyringomyelia (blue arrow) (B and C).

A 30-year-old female patient came with complaints of back pain and neck pain for a year and now worsening of the neck pain with decreased sensation in both upper limbs. She had a history of a vehicular accident 12 months ago. No imaging was done before. An MRI study of the cervical spine with whole spine screening was done. On imaging, the patient had a 12 mm displacement of the cerebellar tonsils in the upper spinal canal across the foramen magnum. Associated HSM was observed that extended from the C2-C3 to the D5-D6 intervertebral disc level with artifacts of the CSF flow due to the disrupted normal CSF flow at the foramen magnum (Figure 1). The patient was operated on for some relief in symptoms and advised for follow-up.

Case 2

**Figure 2:**
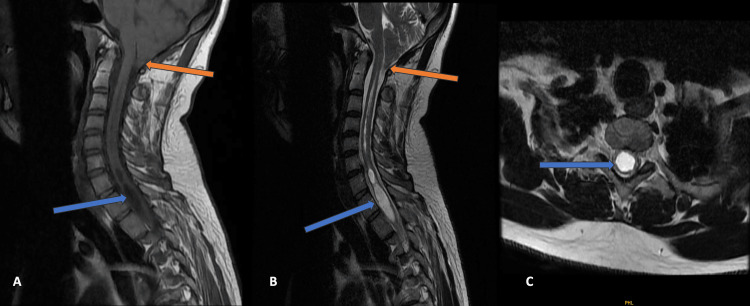
MRI of the cervical spine Magnetic resonance imaging (MRI) of the cervical spine, mid-sagittal T1- and T2-weighted images that show tonsillar herniation (orange arrow) and hydrosyringomyelia (blue arrow) (A and B). An axial T2-weighted MRI image of the cervical spine shows hydrosyringomyelia (blue arrow) (C).

A 34-year-old male patient came with complaints of decreased sensation of pain and temperature on the right side of his neck for six months. She was evaluated with an MRI cervical spine with whole-spine screening. MRI findings revealed a displacement of the cerebellar tonsils in the magnum of the foramen by 11 mm. Associated HSM was seen to extend from the C2 to D9 vertebral levels. The disturbance of the CSF flow and associated flow artifacts were also evident (Figure 2). The patient was advised of decompression surgery.

Case 3

**Figure 3:**
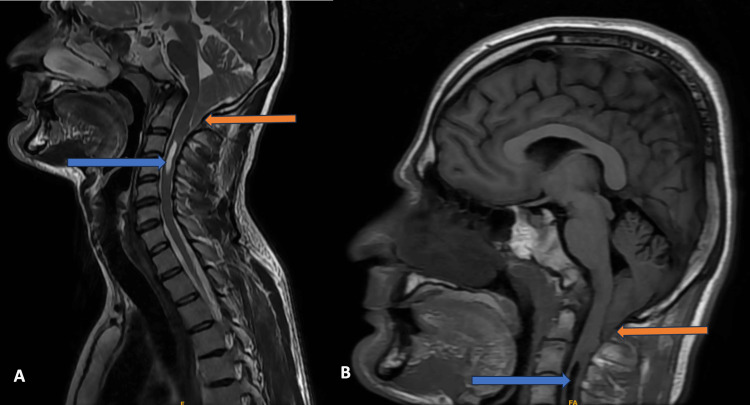
MRI of the cervical spine and brain Magnetic resonance imaging (MRI) of the cervical spine, mid-sagittal T2-weighted image, and MRI of the brain, mid-sagittal T1-weighted image, show tonsillar herniation (orange arrow) and hydrosyringomyelia (blue arrow), appearing hyperintense on T2 and hypointense on T1-weighted images (blue arrow) (A and B).

A 32-year-old male patient came with complaints of headache and neck pain. MRI of the brain and cervical spine was performed. On imaging, the patient had a displacement of the cerebellar tonsils into the upper spinal canal across the foramen magnum by 12 mm. A small syrinx was observed extending from vertebral levels C3 to C4 (Figure 3). The CSF flow artifacts were seen at the level of the foramen magnum and cervical spine. The patient is under observation and is advised to follow up.

Case 4

**Figure 4:**
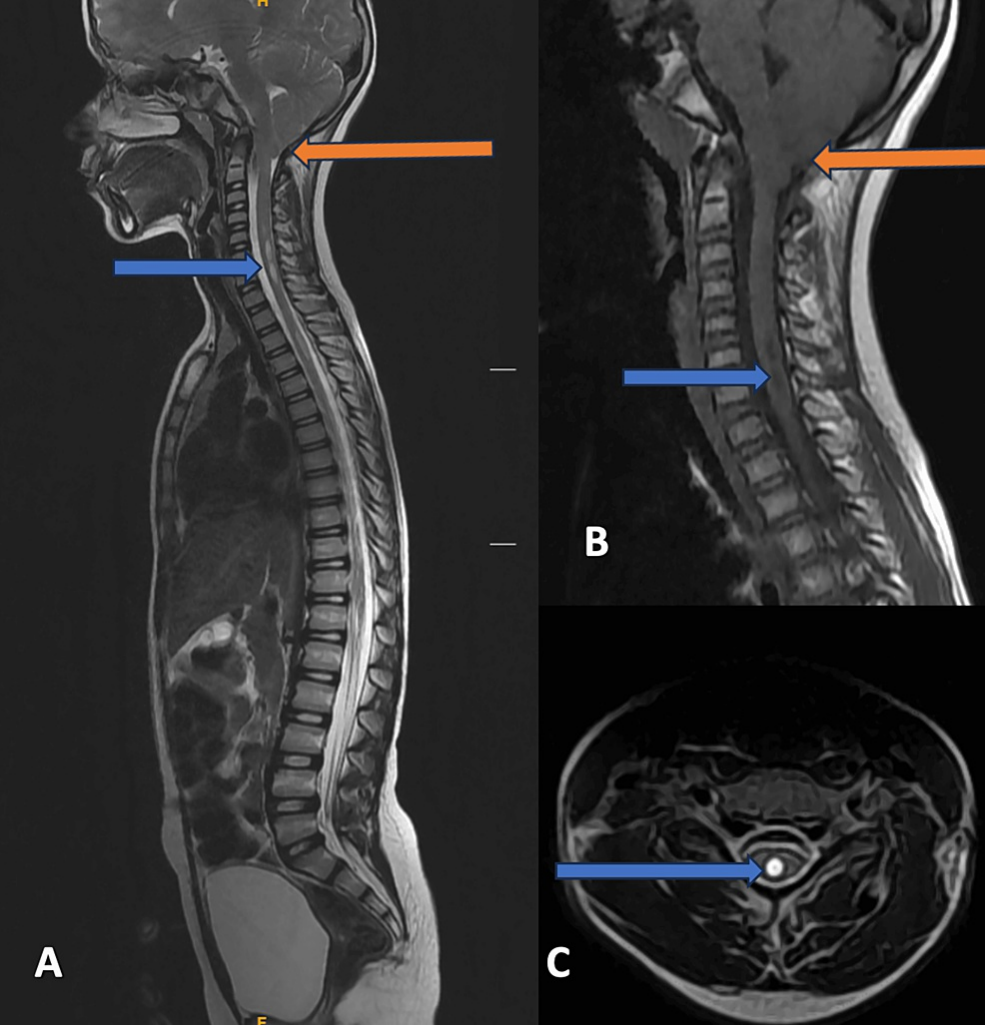
MRI of the cervical spine with whole-spine screening Magnetic resonance imaging (MRI) of the whole spine, mid-sagittal T2-weighted images, and mid-sagittal T1-weighted images of the cervical spine show tonsillar herniation (orange arrow) and hydrosyringomyelia (blue arrow) (A and B). The axial T2-weighted MRI image shows hydrosyringomyelia (blue arrow) (C).

An eight-year-old child came with complaints of decreased pain and temperature sensations in both hands. MRI of the cervical spine with whole-spine screening was advised. MRI revealed displacement of the cerebellar tonsils into the upper spinal canal across the foramen magnum by 9 mm and associated HSM extending from the C5 to D4 vertebral levels (Figure 4). The CSF flow abnormality was also seen at the foramen magnum. The decompression surgery was planned; however, the patient refused the surgery and preferred follow-up.

Case 5

**Figure 5:**
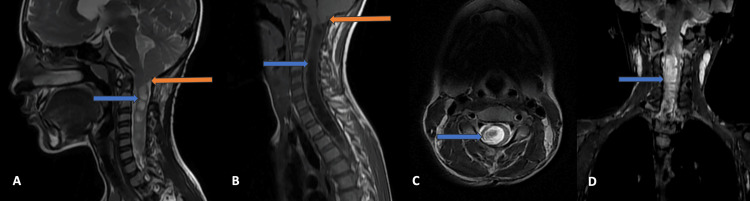
MRI of the cervical spine Magnetic resonance imaging (MRI) of the cervical spine, mid-sagittal T2- and T1-weighted images show tonsillar herniation (orange arrow) and hydrosyringomyelia (blue arrow) (A and B). Axial and coronal T2-weighted MRI images of the cervical spine show hydrosyringomyelia (blue arrow) (C and D).

An eight-year-old boy came with complaints of weakness in all limbs. MRI of the cervical spine was done. On MRI, he had a borderline displacement of the cerebellar tonsils into the upper spinal canal across the foramen magnum by 4 mm. The spinal cord showed extensive changes in HSM involving the entire length, extending from the cervico-medullary junction to the conus medullaris. Associated CSF flow artifacts were also noted (Figure 5). The patient is on a symptomatic treatment.

Case 6

**Figure 6:**
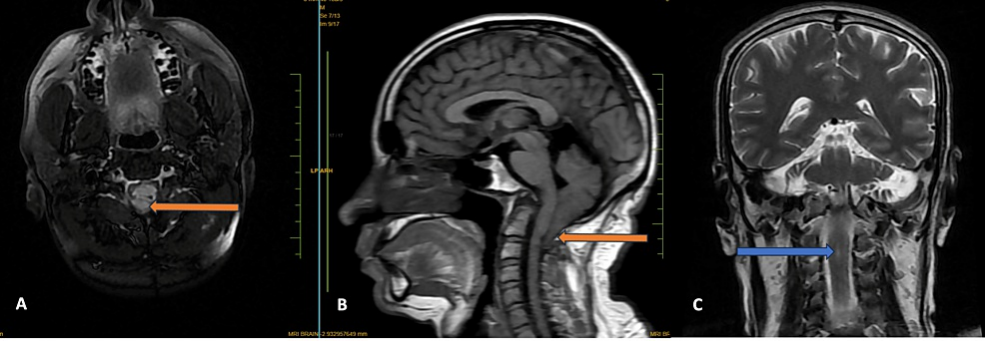
MRI of the brain Magnetic resonance imaging (MRI) of the brain, axial T2 fluid-attenuated inversion recovery (FLAIR), and mid-sagittal T1-weighted images show tonsillar herniation (orange arrow) (A and B). The mid-sagittal T1-weighted image also shows platybasia with an abnormal skull base angle of 147 ° degrees (B). The coronal T2-weighted image shows a small syrinx at the C2-C3 intervertebral disc level (blue arrow) (C).

A 32-year-old male patient came with complaints of headache and neck stiffness. MRI scan of the brain revealed the descent of the cerebellar tonsils into the upper spinal canal by 22 mm. This patient also had flattening of the skull base with an abnormal skull base angle of 147 degrees, suggesting platybasia. A small syrinx was observed at the C2-C3 vertebral level (Figure 6). The rest of the brain parenchyma was normal. The patient refused any surgical treatment and is currently on follow-up. 

Cases 1-4 were classical presentations according to CM1 (Figures 1-4). These were characterized by the descent of cerebellar tonsils greater than 6 mm but less than 12 mm into the upper cervical spinal canal, with varying degrees of syrinx formation. Case 5 had borderline tonsillar herniation of 4 mm and extensive HSM that extended from the cervico-medullary junction to the conus medullaris (Figure 5). It can be considered a variation of CM1 with borderline tonsillar herniation and HSM, or it can be considered a CM0 malformation [15-17]. Case 6 is another end of this spectrum with 22 mm tonsillar herniation with a very small syrinx (Figure 6). This patient also had platybasia, which further complicates the appearance of the imaging [18-20]. It can be considered CM1.5, which can be a separate entity or can be a variation of CM1 [21,22].

## Discussion

CM is a congenital condition that involves abnormalities at the junction of the skull and spine. The Austrian pathologist Hans Chiari first described this condition in the 1890s [2]. Previously, four types of CMs were described, with five more variations added recently according to the symptomatology and imaging appearance; hence, in total, we have nine different types of CMs. CM1 is the most common CM. CMs are often asymptomatic and are frequently discovered incidentally during diagnostic imaging. They can lead to CSF flow disturbances and associated HSM. Nine different types of CMs are described in the literature based on the imaging appearance and severity of the disease (Table 2).

**Table 2:** Types of Chiari malformations CM: Chiari malformation

Sr No.	Type of Chiari malformation (CM)	Findings
1	CM0	A syrinx within the spinal cord without herniation of cerebellar tonsillar or brain stem through the foramen magnum.
2	CM0.5	The ventral tonsillar wrapping around the medulla in the absence of a cerebellar tonsillar descent in the upper spinal canal.
3	CM1	The descent of cerebellar tonsils (more than 6 mm) into the upper cervical spinal canal.
4	CM1.5	The caudal descent of cerebellar tonsils and brainstem through the foramen magnum greater than 12 mm.
5.	CM2	The medulla, fourth ventricle, and cerebellum are pushed downward into the upper cervical spinal canal with lower spinal myelomeningocele.
6	CM3	A high cervical-occipital encephalocele and protruding cerebellum and/or brainstem.
7	CM3.5	An occipitocervical encephalocele communicating with the foregut.
8	CM4	The severe cerebellar hypoplasia without displacement of the cerebellum through the foramen magnum.
9	CM5	The absent cerebellum with occipital lobe herniation through the foramen magnum.

CM0 is defined as a syrinx within the spinal cord without herniation of the cerebellar tonsillar or brain stem through the foramen magnum. However, it is not widely used and might be misleading, so it should be avoided. Improvement in the syrinx has been documented after posterior fossa decompression [15-17]. CM0.5 is distinguished by the ventral tonsillar wrapping around the medulla in the absence of a cerebellar tonsillar descent in the upper spinal canal as in classic CM1 malformation [23]. CM1 is distinguished by the descent of cerebellar tonsils (more than 6 mm) into the upper cervical spinal canal. It is related to syringomyelia and hydromyelia. Adolescents and adults are more likely to be diagnosed with this type. It may be associated with skull and spinal malformations [10-14].

CM1.5 is documented in the literature as both a separate disorder and a variant of CM1 malformation. Caudal descent of cerebellar tonsils and brainstem greater than 6 mm suggests CM1 malformation, while a descent greater than 12 mm indicates CM1.5 malformation [21,22]. CM2 is a condition in which the medulla, fourth ventricle, and cerebellum are pushed downward into the cervical spinal canal, which is almost always seen in patients with lower spinal myelomeningocele. It is related to a variety of congenital defects [24-25]. CM3 is characterized by a high cervical-occipital encephalocele and the cerebellum and/or brainstem, protruding through a hole in the back of the head or neck. It is a rare condition with severe neurological impairments and a high early mortality rate [26].

Muscatello documented a case of an occipitocervical encephalocele communicating with the foregut [24,27]. He suggested that this anomaly be referred to as CM3.5. It was not compatible with life [24,27]. CM4 is the most severe and rare type of CM that involves faulty cerebellar development. Severe cerebellar hypoplasia is seen without displacement of the cerebellum through the foramen magnum. This condition is frequently accompanied by other brain and brainstem abnormalities [3,24]. The absent cerebellum with occipital lobe herniation through the foramen magnum is the hallmark of this very rare condition described as CM5 [24,28].

Symptoms vary according to the type and severity of the malformation. Common symptoms include headache, neck pain, sensory and motor deficits, dizziness, and balance problems. In children, symptoms can be subtle and nonspecific, such as motor delays or swallowing difficulties [6,7].

The preferred diagnostic method for CM1 and HSM is MRI. Computed tomography scans are important for identifying bony anomalies and for rapid postoperative assessment. Additional tests, such as sleep studies, swallowing studies, and auditory evoked potential tests, can also be performed [10-14].

In CM1, axial and sagittal T1- and T2-weighted fast spin-echo sequences are minimally required. However, for a comprehensive evaluation with CSF flow study, the MRI protocol should include axial and sagittal T1- and T2-weighted fast spin-echo sequences, sagittal cardiac-gated phase-contrast cine-mode images, sagittal cardiac-gated cine true fast imaging with steady-state precession (true FISP), and sagittal high-spatial-resolution cisternography sequences. In addition, STIR, contrast-enhanced T1-weighted sequences, and diffusion-weighted (DW) pictures are helpful for suspected acute infarcts or infections. Heavily T2-weighted MR cisternography-type sequences, such as 3D-driven equilibrium (DRIVE), fast imaging employing steady-state acquisition (FIESTA), or constructive interference in steady state (CISS), can provide precise delineation of parenchyma-CSF interfaces, thus allowing for a detailed assessment of the cerebellar morphology [29].

We have conducted a preliminary study for diagnosing patients with CM1 using the T1-weighted and T2-weighted images in the axial and sagittal planes, T2-weighted gradient echo images in the sagittal plane, and STIR images in the sagittal and coronal planes. A qualitative assessment of the CSF flow disturbances was noted on T2- and T2-weighted gradient echo images in the sagittal plane. The dynamic CSF flow study was not done.

MRI allows for high-resolution imaging of the brain and spinal cord, providing detailed visualization of anatomical structures. In CM1, MRI helps identify the characteristic downward displacement of the cerebellar tonsils through the foramen magnum. In addition, MRI can reveal associated abnormalities, such as overcrowding of the posterior fossa, cerebellar herniation, and compression of the brainstem. The cerebellar tonsils are positioned below the level of the foramen magnum (>3-5 mm), often compressing the brain stem and impeding CSF flow. MRI allows for accurate measurement of tonsillar descent, which is essential for diagnosing CM1 and grading its severity. Tonsillar descent measurements are typically obtained by assessing the distance between the tip of the cerebellar tonsils and the basion (a skull landmark) on sagittal MRI images. These measurements help classify CM1 into different severity categories and guide treatment decisions [8-9,12-14].

MRI is indispensable for detecting and characterizing syrinx formation in HSM. It enables radiologists to assess the size, shape, and extent of the syrinx and its relationship with surrounding neural structures. In cases of HSM, MRI aids in detecting the presence of fluid-filled cavities (syrinx) within the spinal cord and assessing their size, location, and extent. The syrinxes within the spinal cord appear as fluid-filled cavities with CSF-like signal intensity on AI sequences. They can extend over multiple spinal segments, and their size and location can vary. The syrinxes are often elongated and cantered in the central region of the spinal cord, leading to characteristic "butterfly" or "hourglass" shapes. Syringomyelia and hydromyelia are two closely associated conditions that cannot be distinguished from each other on imaging, hence termed HSM [8,13]. Dynamic MRI sequences, such as cine flow studies and phase-contrast imaging, provide insights into CSF dynamics at the craniocervical junction. Abnormal CSF flow patterns, including obstruction or turbulence, can contribute to the pathogenesis of CM1 and HSM. This disruption in flow dynamics is associated with the development and progression of syringomyelia. T2-weighted images are particularly useful in visualizing syrinxes within the spinal cord. MRI helps to identify CSF flow abnormalities, which are crucial for surgical planning and predicting postoperative outcomes [8,9].

MRI plays a vital role in guiding treatment decisions and monitoring disease progression in patients with CM1 and HSM. It helps neurosurgeons in preoperative planning by identifying relevant anatomical landmarks, optimizing surgical approaches, and predicting surgical outcomes. Serial MRI examinations are essential for postoperative follow-up to assess the efficacy of surgical interventions, monitor syrinx regression, and detect potential complications.

Treatment depends on the type, symptoms, and progression of the malformations. Asymptomatic CM1 is usually left untreated. Symptomatic cases, or those with syringomyelia, may require surgery. Surgery aims to decompress nerve tissue and restore normal CSF flow. Follow-up MRI examinations can be useful for evaluating these patients after surgery and assessing potential surgical complications to provide timely and appropriate diagnoses. Some patients may require repeat surgeries [29,30].

All symptomatic patients were advised decompression surgery; out of the six patients, one got operated on and got relief from sensory symptoms postoperatively. One patient was advised for surgery; however, he failed to follow up. Two patients refused to undergo surgery. Two patients with milder symptoms of headache and neck pain/neck stiffness are under observation and on symptomatic treatment.

The limitations of this study include its retrospective design, potential selection bias, and reliance on available medical records for data collection. In addition, variability in MRI protocols and image quality may have influenced the accuracy of imaging findings.

## Conclusions

MRI is the modality of choice for confirming the diagnosis of CM1. It also helps determine the severity of the malformations and identify any additional complications, such as HSM. This information is crucial for treatment planning and management decisions. MRI scan helps surgeons visualize the extent of the malformation and provide a road map for planning the surgical procedure accordingly.
